# Dynamics of *Toxoplasma gondii* Oocyst Phagocytosis by Macrophages

**DOI:** 10.3389/fcimb.2020.00207

**Published:** 2020-05-19

**Authors:** Omar Ndao, Pierre-Henri Puech, Camille Bérard, Laurent Limozin, Sameh Rabhi, Nadine Azas, Jitender P. Dubey, Aurélien Dumètre

**Affiliations:** ^1^Aix Marseille Univ, IRD, AP-HM, SSA, VITROME, Marseille, France; ^2^IHU-Méditerranée Infection, Marseille, France; ^3^Aix Marseille Univ, LAI UM 61, Marseille, France; ^4^Inserm, UMR_S 1067, Marseille, France; ^5^CNRS, UMR 7333, Marseille, France; ^6^Animal Parasitic Diseases Laboratory, Beltsville Agricultural Research Center, United States Department of Agriculture, Agricultural Research Service, Beltsville, MD, United States

**Keywords:** *Toxoplasma gondii*, oocysts, sporozoites, excystation, macrophages, phagocytosis, optical tweezers, micropipette aspiration techniques

## Abstract

Oocysts are the environmentally resistant stage of the protozoan parasite *Toxoplasma gondii*. They are responsible for foodborne infections in humans and animals worldwide. Infectious oocysts contain sporozoites that have to exit the sporocyst and oocyst walls to initiate replication of the parasite within the host tissues. Given their robustness and resistance to chemical degradation, it is still unclear how the oocyst and sporocyst walls release the sporozoites. This process called excystation is thought to occur in the small intestine as a result of the combined action of digestive agents, yet to be identified. By using an oocyst-macrophage co-culture platform, we previously demonstrated *in vitro* that the excystation of sporozoites and their differentiation into replicative tachyzoites could occur in absence of digestive factors, following phagocytosis by macrophages. Here, we further characterize the dynamics of the oocyst phagocytosis at the single-cell level by using optical tweezers and micropipette aspiration techniques. Our results show that the oocyst internalization kinetics can vary among a given population of macrophages, but similar processes and dynamics could be observed. Most of the cells manipulate oocysts for ~15 min before internalizing them in typically 30 min. This process mainly involves the actin cytoskeleton of the macrophages. Liberated sporozoites within macrophages then differentiate into tachyzoites within 4–6 h following oocyst-macrophage contact. Tachyzoites appear to develop better in macrophages challenged with free sporocysts or sporozoites than with whole oocysts, suggesting that opening of the oocyst wall is one of the most limiting steps for sporozoite excystation completion.

## Introduction

The apicomplexan parasite *Toxoplasma gondii* can persist throughout the environment as a robust infectious stage called the oocyst (Shapiro et al., 2019). Oocysts are excreted in cat feces and become infectious following a 1–2 week sporulation process. Sporulated oocysts measure ~13 × 11 μm and contain two sporocysts, each with four potential infective sporozoites that are protected from harsh environmental conditions by the sporocyst and oocyst walls (Freppel et al., 2019). Oocysts can infect many avian and mammal species worldwide, including humans, through the consumption of water or raw vegetables and fruits contaminated with cat feces (Shapiro et al., 2019). Following ingestion, sporozoites excyst from the sporocyst and oocyst walls, invade host enterocytes, and lamina propria macrophages and dendritic cells prior to differentiation into tachyzoites (Delgado Betancourt et al., 2019). Tachyzoites can replicate within these phagocytic cells, and use them as Trojan horses to disseminate throughout the body (Drewry et al., 2019). Infection results in the development and persistence of the parasite as tissue cysts, mainly in the brain and muscles. In turn, tissue cysts in undercooked meat can be a source of human contamination. Irrespective of the ingested stage, most infections are asymptomatic except in congenitally infected children and immunocompromised people, who may suffer severe ocular, cerebral, or multivisceral complications (Robert-Gangneux and Dardé, 2012).

The *T. gondii* oocyst and sporocyst walls are bilayered structures, mainly composed of proteins (Freppel et al., 2019). The outer oocyst wall layer contains cysteine- and tyrosine-rich proteins that form extensive disulphide bridges and dityrosine cross-links, respectively, and triglycerides that are similar to mycobacterial mycolic acids. The inner oocyst wall layer consists of cross-linked Tyr-rich proteins and scaffolds of beta-1,3-glucan. The outer sporocyst wall layer is similar to the outer oocyst wall layer in structure while its inner layer is made of four curved plates held together by thick sutures. The sporocyst wall resembles the oocyst wall in molecular composition, except that it lacks cysteine-rich proteins and beta-1,3-glucan. Both walls are naturally blue fluorescent under UV excitation, presumably due to their dityrosine cross-links. Due to their structure and molecular composition, the oocyst and sporocyst walls appear very resistant to mechanical constraints and enzymatic digestion, and almost impermeable to water-soluble substances including common chlorinated disinfectants (Dumètre et al., 2013).

Given their robustness and resistance to chemical degradation, it is still unclear how the oocyst walls open to allow the sporozoites to invade the host cells. Ingested oocysts release their sporozoites in the small intestine, however the digestive agents that trigger the opening of the oocyst walls are not identified. Interestingly, oocysts can cause parenteral infections, at least in laboratory mice, suggesting a possible excystation of sporozoites in absence of digestive factors (Dubey and Frenkel, 1973). From these observations, we developed *in vitro* oocyst-macrophage co-cultures to investigate whether phagocytic cells could mediate sporozoite excystation following oocyst phagocytosis (Freppel et al., 2016). Previous *in vitro* experiments showed that naïve RAW 264.7 macrophagic cells could ingest oocysts, open their walls in or near acidic compartments, and host the differentiation of the sporozoites into replicative tachyzoites. In the present study, we extend the use of this oocyst-macrophage co-culture platform to further characterize the dynamics of the oocyst internalization at the single-cell level and the fate of the sporozoites within macrophages. We used optical tweezers and micropipettes to present oocysts to living macrophages, either adherent or not, at different incubation temperatures. Our results show that most of cells manipulate oocysts for ~15 min before internalizing them in ~30 min, by remodeling their actin cytoskeleton. Liberated sporozoites within macrophages then differentiate into tachyzoites within 4–6 h following oocyst-macrophage contact. Tachyzoites appear to develop better in macrophages challenged with free sporocysts or sporozoites than with whole oocysts suggesting that opening of the oocyst wall is one of the most limiting steps for sporozoite excystation completion in macrophages.

## Materials and Methods

### Macrophage Cell Culture Conditions

Mouse macrophage-like cell line RAW 264.7 was purchased from European Collection of Authenticated Cell Cultures (ECACC, Salisbury, United-Kingdom). Cells were cultured at 37°C and 5% CO_2_ in plastic 75-cm^2^ flasks containing RPMI 1640 medium (Life Technologies, Saint-Aubin, France) supplemented with 2 mM L-glutamine, 100 U/ml penicillin, 100 μg/ml streptomycin (Life Technologies), and 10% heat-inactivated fetal bovine serum (FBS) (Life Technologies). Cells reaching ~80% confluency were detached with a cell scraper and subcultured following a 10-fold dilution in fresh culture medium. Before parasite internalization assays, cells were detached as described, centrifuged at 400 g for 10 min, counted on Kova® slides and their concentration was adjusted in fresh culture medium.

### Oocyst Production and Purification

Oocysts of the reference genotypes II Me49 or III VEG strains of *T. gondii* were used throughout this study and were manipulated in biosafety level 2 facilities. Available bioinformatics data indicate that oocysts of both genotypes have very similar predicted transcriptome and proteome (http://toxodb.org/toxo/). Oocysts were harvested from feces of cats 3–10 days after feeding infected mouse tissues to *T. gondii* free cats as described previously (Dumètre et al., 2013). This procedure was carried out in accordance with relevant guidelines and regulations following a protocol approved by the Institutional Animal Care and Use Committee, United States Department of Agriculture, Beltsville, MD, USA. Oocysts were collected by flotation at 4°C from cat feces on a 1.15 density sucrose solution, washed in distilled water and then resuspended in an aqueous solution containing 2% H_2_SO_4_. Oocysts were allowed to sporulate at room temperature (RT, 20–22°C) for 5 days under adequate aeration and gentle continuous shaking. For real-time internalization assays by using optical tweezers and micropipette aspiration, oocysts were further purified on a cesium chloride gradient to remove most of fecal debris (Dumètre and Dardé, 2004). Oocysts were stored in a 2% H_2_SO_4_ aqueous solution at 4°C until used. Prior experiments, oocysts were washed three times in sterile distilled water at 10,000 g for 2 min and once in cell culture medium. Oocyst concentration was adjusted by counting the parasites on Kova® slides under bright field on an Olympus BX51 equipped with a 40x objective.

### Trapping Oocysts With Optical Tweezers for Internalization Assay Using Adherent Macrophages at 37°C

We used optical tweezers coupled to real-time imaging to characterize the dynamics of the oocyst internalization by adherent macrophages at 37°C. The experiments were carried out on the NanoTracker™ 2 (JPK instruments) platform, which includes an inverted microscope (Carl Zeiss, Axio Observer) equipped with a motorized plate, CCD camera, and an infrared laser source (wavelength 1,064 nm). The microscope is equipped with a motorized piezoelectric stage that allows the sample to be observed in all three directions (x, y, z). The movements, laser, and detection are controlled under GNU/Linux using NanoTrackerTM JPK Instrument software, which contains JUnicam frame grabber for image capture. A thermoregulator (JPK Petri Dish holder) allows the temperature of the sample to be maintained at 37°C during the measurement.

Petri dishes with 35 mm in diameter and 0.17 mm thick glass bottom (FluoroDish®, WPI) were used as observation chambers. A volume of 10 μl of macrophage cells (i.e. ~1.5.10^5^ cells) in 2 ml fresh culture medium was placed in the dish in order to obtain a homogeneous dispersion of the cells. Macrophages were incubated overnight at 37° C and 5% CO_2_ to allow them to adhere. After two successive washes in PBS, 2 ml of pre-warmed culture medium were deposited onto the cells. Oocysts in 10 μl culture medium were added at ratio 1:1 and let for sedimentation for about 5 min before performing the experiments.

**Figure 1:**
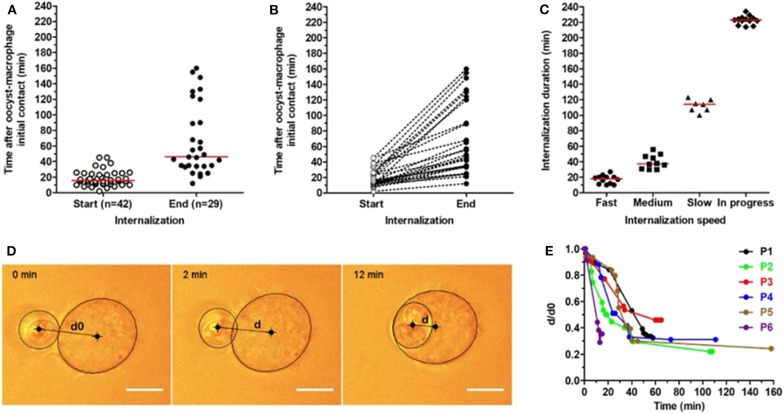
Kinetics of *T. gondii* oocyst internalization by RAW macrophages measured using optical tweezers. Oocysts were trapped by optical tweezers and individually presented to macrophages. Each oocyst-macrophage pair was then followed for oocyst internalization by real-time imaging at 37°C for a maximum 240-min period. **(A)** Start and end of oocyst internalization after oocyst-macrophage initial contact (t0). The numbers of oocyst-macrophage pairs for which oocyst internalization started (*n* = 42) and ended (*n* = 29) within the 240-min period are indicated. Each dot represents an oocyst-macrophage pair. The red line represents the median of data distribution. **(B)** Distribution of oocyst-macrophage pairs (*n* = 29) that completed oocyst internalization during the period and **(C)** the internalization speed. Speed was arbitrarily classified as fast (full internalization in 0–29 min), medium (30–59 min), slow (≥60 min), and still in progress at the end of the experiments. Each symbol represents an oocyst-macrophage pair. The red line represents the median of data distribution. **(D,E)** Oocyst internalization dynamics was further characterized in 6 oocyst-macrophage pairs (P1 to P6) by quantifying d/d0 up to 160 min following contact, with d0 the apparent distance measured between the oocyst and macrophage centers at t0 (initial contact) and d the apparent oocyst-macrophage distance at *t* > 0. A zoom on the 0–60 min period is available in the Supplementary Figure 1. Scale bar = 10 μm.

An observation area containing several adhered macrophages was first chosen. An oocyst was then trapped with the laser and moved to the defined area to make slight contact with a macrophage (Supplementary Movie 1). After 10 sec of oocyst-macrophage contact, the laser was switched off to release the oocyst. An image was taken just after releasing the oocyst (*t* = 0, see an example in Figure 1D). The same process was repeated until a free oocyst was presented to each of the macrophages present in the defined area. A second zone was then selected and so on in order to have enough oocyst-macrophage pairs for statistical analyses. Then, oocyst-cell interactions were monitored, zone-by-zone, over a minimum duration of 70 min and up to 4 h, with a time step of 5 min sufficient to find the desired zone and perform a multiple acquisitions in z using the piezo stage to assess that oocysts were within the macrophage cells.

Images were analyzed using the Fiji/ImageJ software (https://fiji.sc/) to quantify the beginning and end of oocyst internalization for each oocyst-macrophage pair. We defined the beginning of oocyst internalization when macrophages started producing cellular extensions stretching toward oocysts and the end at the complete engulfment of the parasite as assessed by z scan map acquisition. Oocyst internalization was also quantified by recording the positions of the macrophage and the oocysts by elliptical/oval overlays and measuring the distance between the center of the macrophage and the center of the oocyst as a function of time (Figure 1D).

### Micropipette Aspiration for Internalization of Oocysts by Non-adherent Macrophages at Room Temperature

We used the micropipette aspiration technique described by Freppel et al. (2016). Briefly, a glass capillary (internal diameter 0.58 mm/outer diameter 1 mm) was heated using a micropipette puller (David Kopf Instruments, Model 700C) until melting, and separated in half to obtain two closed microneedles. The tips of the two microneedles were forged as micropipettes under microscope (Microforge, Alcatel) until the desired shape and size of the opening were obtained (~2–3 μm). The micropipettes were then bent with a 30°-angle using a second microforge (Microforge MF-830, Narishige). Both micropipettes were filled carefully with PBS/1% BSA medium to passivate their inner walls. The observation chamber consisted in a Petri dish with 35 mm in diameter and 0.17 mm thick glass bottom (FluoroDish®, WPI) filled with 2 ml of culture medium (RPMI 1640 + 10% FBS). The micropipettes were introduced in the chamber to passivate their outer wall 5–10 min before the experiment. They were mounted on x,y,z micromanipulators (one Narishige hydraulic system, one Sutter Instruments MP285 electronic one), facing each other on an inverted microscope Olympus IMT2, equipped with a Prosilica GE680 CCD camera for fast acquisitions under bright field at 40x magnification.

Aspiration of cells in suspension was performed after exchange of the buffer with culture medium at RT, introduction of diluted cells and parasites and their sedimentation. Then, a non-adherent macrophage and one oocyst were selected and aspirated with each micropipette using a homemade aspiration system based on interconnected vessels. The aspiration pressure was monitored using pressure sensors (typically −2/−3 cm H_2_O for the macrophage, and −2/−10 cm H_2_O for the oocyst). The oocyst was presented and gently pressed to the macrophage for 1 min. Aspiration was then quickly released on the oocyst, and the holding pipette removed while the oocyst/macrophage pair was observed over time for 60 min maximum. Images were acquired using a homemade software programmed in Python 3.6 or using proprietary acquisition Vimba from Allied Vision (https://www.alliedvision.com/en/products/software.html), and analyzed using Fiji/ImageJ software. Multiple acquisitions in z were performed by changing lens focus to assess that the oocyst was internalized by the macrophage. The culture medium, micropipettes and oocyst/macrophage suspensions were changed every hour. Experiments were run at RT.

### Inhibition of Oocyst Internalization

Macrophages were plated in triplicate at the density of 1.5.10^5^ cells in 1 ml culture on 12 mm-glass coverslips (0.13–0.17 mm thick) precoated with 0.01% poly-L-lysine solution placed at the bottom of 24-well sterile plates as described previously (Freppel et al., 2016). After incubation for 24 h at 37°C, cells were pre-treated for 30 min with 1 μM of the actin polymerization inhibitor latrunculin A (LatA, Sigma-Aldrich, Saint-Quentin-Fallavier, France) or 50 μM of the myosin-II inhibitor blebbistatin (Blebb, Sigma-Aldrich). Oocysts were added at ratio 1:1 and incubated with macrophages at 37°C for 1 h in continuous presence of 1 μM LatA or 50 μM Blebb. Cells incubated with 0.1% DMSO served as carrier control. After that, coverslips were washed three times with PBS, fixed in 4% (w/v) paraformaldehyde (PFA) in PBS for 15 min at 4°C, rinsed three times with PBS, and then permeabilized with 0.1% TritonX-100 in PBS for 15 min at RT. After three washes in PBS, coverslips were incubated for 40 min at RT on a drop of rhodamine-conjugated phalloidin (Life Technologies) diluted at 1:40 in PBS for observation of actin filaments. After three washes in PBS, coverslips were air-dried and mounted with SlowFade® Gold Antifade DAPI mounting medium (Life Technologies). Bright field and epifluorescence images of fixed cells were collected on an Olympus BX51 equipped with a XC30 CCD camera, 40x and 100x objectives, and epifluorescence filters for DAPI and rhodamine. Images were acquired using the imaging system CellA (Olympus) and were further analyzed using the Fiji/ImageJ software. Macrophages with oocysts attached at their surface, or partially or fully internalized were counted manually over randomly selected 20–40 fields (~150–200 macrophages) per coverslip and condition.

### Detection of the Sporozoites and Tachyzoites in Oocyst-Macrophage Co-cultures

Macrophages were plated on coverslips as described above and co-incubated with oocysts at ratio 1:1 for 4, 6, or 24 h at 37°C. After that, preparations were fixed in absolute cold methanol for 10 min and rinsed three times in PBS. They were then incubated at 37°C for 1 h with a human antiserum reacting with both *T. gondii* sporozoites and tachyzoites at 1:200 dilution in PBS and a rabbit polyclonal sporozoite-specific anti-AMA4 antibody at 1:1000 dilution in PBS. After three washes in PBS, cells were then incubated at 37°C for 1 h with a FITC-conjugated secondary anti-human antibody at 1:100 dilution in PBS and an Alexa546-conjugated secondary anti-rabbit antibody at 1:50 dilution in PBS. After three washes in PBS, coverslips were air-dried, mounted with SlowFade® Gold Antifade DAPI mounting medium, and observed for sporozoite and tachyzoite detection using the Olympus BX51 set-up. The respective number of sporozoites and tachyzoites was determined by manually counting the parasites over ~200 macrophages per coverslip.

### Monitoring the Development of Tachyzoites in Macrophages Challenged With Oocysts, Free Sporocysts, or Sporozoites

To investigate whether removal of the oocyst and sporocyst walls modifies the development of tachyzoites from sporozoites over time, macrophages were challenged either with oocysts, free sporocysts or sporozoites. Sporocysts and sporozoites were obtained following oocyst sonication and further incubation in an excystation medium as detailed by Freppel et al. (2016). Macrophages (1.5.10^5^ cells) were plated on coverslips as described above and challenged with oocysts (ratio 1:1), freshly released sporocysts or freshly excysted sporozoites in cell culture medium. Sporocysts were added at ratio 2:1 assuming that one fully sporulated oocyst contained two sporocysts. Sporozoites were added at a quantity equivalent to 1.5 × 10^5^ oocysts, i.e., ~1.2 × 10^6^ sporozoites assuming that one fully sporulated oocyst contained eight sporozoites. Macrophages and parasites were co-cultured for 24 h at 37°C to allow the development of tachyzoites. After that, coverslips were processed as described above for tachyzoite detection by fluorescence microscopy. The percent of macrophages containing tachyzoites was determined by manually counting ~200 macrophages per coverslip and condition.

### Statistical Analyses

All data were analyzed by using GraphPad Prism 5.03 software. Statistical significance between the data sets was evaluated by one-way ANOVA tests followed by the Tukey's multiple comparison test. *P*-values <0.05 were considered significant: ^***^, ^**^, and ^*^ indicate *p* < 0.001, *p* < 0.01, and *p* < 0.05, respectively. Unless stated, data were expressed as mean ± standard deviation (S.D.) of four independent experiments.

## Results

### Oocysts Are Internalized by Macrophages at Different Speeds

To study the dynamics of oocyst internalization at the single-cell level, we used optical tweezers for trapping oocysts and presenting them individually to macrophages (see an example in Supplementary Movie 1). Following a 10-sec oocyst-macrophage contact, the laser was cut off and each oocyst-macrophage pair (*n* = 46) was followed for 240 min at 37°C. Oocyst internalization started in 42 (91.3%) out of 46 pairs between 2 and 45 min (median 15.5 min) after initial contact (Figure 1A). It ended in 29 (69.0%) out of these 42 pairs between 12 and 160 min (median 46.0 min) after initial contact (Figure 1A). Internalization kinetics varies greatly among oocyst-macrophage pairs (Figures 1B,C). Full internalization was classified as fast (0–29 min, median 18 min, *n* = 12 pairs), medium (30–59 min, median 37.5 min, *n* = 10), and slow (≥60 min, median 114 min, *n* = 7). Several macrophages (*n* = 13) were still internalizing their oocyst at the end of experiments (i.e., at *t* = 240 min after the oocyst-macrophage initial contact) (denoted as ‘in progress' in Figure 1C). Oocyst internalization kinetics was further characterized in 6 oocyst-macrophage pairs by measuring the distance between the oocyst center and the macrophage center at *t* = 0 (d0) and then over time (d) (Figures 1D,E). The ratio d/d0 decreased rapidly by 15 min following contact and did not vary after 40–45 min (Figure 1E and Supplementary Figure 1). Micropipette aspiration experiments showed that oocyst internalization could occur at RT, as at 37°C, in a very few oocyst-macrophage pairs (Supplementary Movie 2, Supplementary Figures 2, 3).

### The Complete Internalization of Oocysts Requires Actin Polymerisation of the Macrophage Cell Cytoskeleton

**Figure 2:**
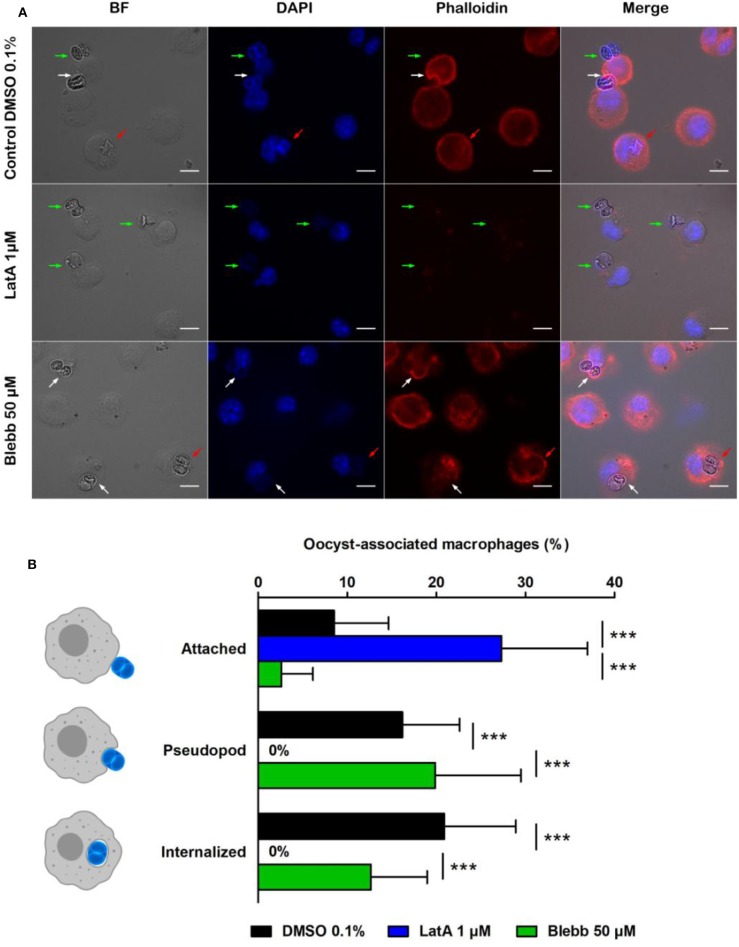
Interactions of *Toxoplasma gondii* oocysts with RAW macrophages in the presence of inhibitors of actin polymerization (latrunculin A, LatA) or myosin II (blebbistatin, Blebb). **(A)** Microscopic monitoring showing macrophages with oocysts attached to their membrane (green arrow), engulfing the parasite (white arrow) or having internalized it (red arrow). BF, bright field; DAPI channel, natural fluorescence of oocysts and macrophage nucleus; phalloidin, macrophage actin cytoskeleton. Scale = 10 μm. **(B)** Percentage of macrophages with an oocyst attached to their membrane, engulfing the parasite (pseudopod stage), or having completely internalized it after 1 h of treatment with 1 μM LatA, 50 μM Blebb, or 0.1% DMSO (control). The values correspond to the mean ± standard deviation of 4 independent experiments. One-way ANOVA and Tukey's post-test, ***p < 0.001.

After 1 h incubation, in control conditions (DMSO 0.1%), 8.5% of macrophages had at least one oocyst attached to their membrane, 16.2% formed pseudopods around oocysts, and 20.9% contained oocysts (Figure 2B). In contrast, treating macrophages by the actin inhibitor LatA (1 μM, 1 h) completely abolished the formation of pseudopods (Figures 2A,B) and thus prevented the internalization of oocysts that remained stuck to the surface of macrophages (Figure 2A). Treating cells with myosin-II inhibitor Blebb (50 μM, 1 h) had no significant effect neither on the pseudopod formation nor on the complete internalization of the oocysts compared to control conditions (Figures 2A,B).

### Differentiation of Sporozoites Into Tachyzoites and Dissemination of Tachyzoites

**Figure 3:**
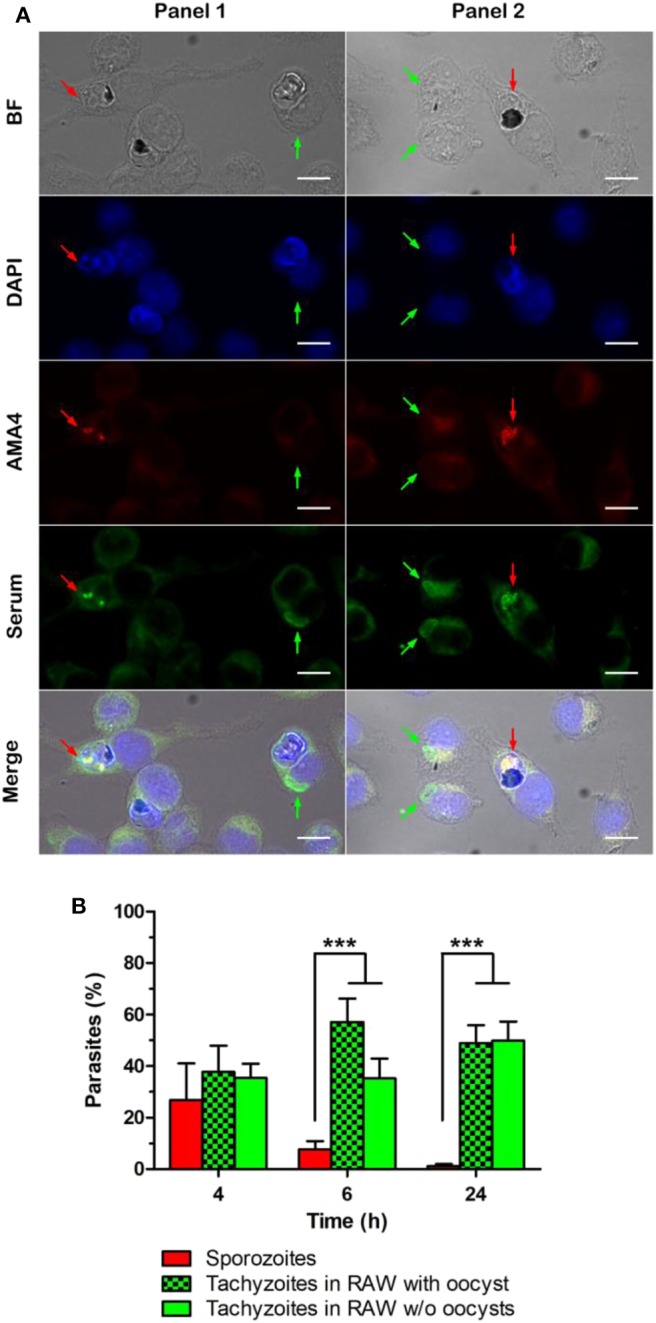
Detection of sporozoites and tachyzoites of *Toxoplasma gondii* after 4, 6, and 24 h of oocyst-RAW macrophage co-incubation**. (A)** Representative example of the immunolabelling of sporozoites (red arrow) and tachyzoites (green arrow) whether present in macrophages with oocyst remnants (Panel 1) or in macrophages without visible oocyst remnants (Panel 2) after 4 h of oocyst-macrophage co-incubation. DAPI, macrophage nucleus and natural fluorescence of oocysts; AMA4, sporozoites; serum, sporozoites and tachyzoites. Scale = 10 μm. **(B)** Percentage of sporozoites (red bar) and tachyzoites [whether present in macrophages with visible oocyst remnants (green and black checker) or in macrophages without oocysts (green)]. Mean ± standard deviation of 3 independent experiments. One-way ANOVA and Tukey's post-test, ***p < 0.001.

After 4, 6, or 24 h of oocyst-macrophage co-incubation, sporozoites and tachyzoites were detected by immunofluorescence using a *T. gondii* positive human serum and a sporozoite-specific AMA4 polyclonal antibody (Figure 3A). Tachyzoite number increased as sporozoite number decreased over time (Figure 3B). Most of tachyzoites appeared to develop from sporozoites within 4 to 6 h after the oocyst-macrophage contact. Irrespective of the time, tachyzoites were almost exclusively observed as single parasites in macrophages whether hosting an oocyst (Figure 3A, panel 1) or not (Figure 3A, panel 2).

### Tachyzoites Develop Better in Macrophages Challenged With Free Sporocysts or Excysted Sporozoites Rather Than Oocysts

**Figure 4:**
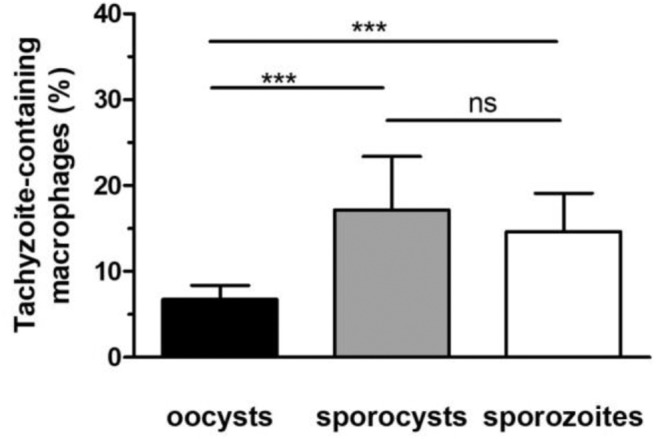
Development of tachyzoites in RAW macrophages challenged with oocysts, free sporocysts, or sporozoites. Following parasite-macrophage incubation for 24 h, the percentage of tachyzoite-containing macrophages was determined by immunofluorescence assay as described in materials and methods. Mean ± standard deviation of 4 independent experiments. One-way ANOVA and Tukey's post-test, ***p < 0.001.

To investigate whether removal of the oocyst and sporocyst walls modifies the development of tachyzoites from sporozoites over time, macrophages were challenged either with whole oocysts, free sporocysts or sporozoites. After 24 h of co-incubation, immunolabelling was performed with antitoxoplasmic positive serum to determine the percentage of macrophages infected with tachyzoites (it was considered that at 24 h the proportion of sporozoites was negligible, see Figure 3B). Following incubation with whole oocysts, 6.8% of macrophages were infected vs. 17.2% with free sporocysts and 14.6% with free sporozoites (Figure 4). Tachyzoites developed better in macrophage cultures challenged with free sporocysts or sporozoites than with whole oocysts (*p* < 0.001) (Figure 4).

## Discussion

How *T. gondii* sporozoites excyst from the oocyst to infect host tissues remains poorly characterized. In particular, host and parasite factors that trigger the opening of the oocyst and sporocyst wall are currently unknown. Oocysts entering the host gut face various digestive agents such as pepsin, trypsin and biliary salts, however none of them (alone or in combination) significantly affect the integrity of the robust oocyst wall (Freppel et al., 2019). Digestive factors even seem unnecessary to the excystation process as *T. gondii* oocysts can cause, in rare conditions, infection following parenteral exposure (Dubey and Frenkel, 1973; Dubey, 2006). We recently hypothesized that, in absence of digestive factors, phagocytic cells could facilitate excystation of the sporozoites *in vitro* following oocyst phagocytosis (Freppel et al., 2016). In the present study, we further characterized the dynamics of the early interactions between oocysts and RAW264.7 macrophages and analyzed the fate of excysted sporozoites in oocyst-macrophage co-cultures.

Micropipette aspiration and optical tweezers allowed us to investigate early interactions between oocysts and non-adherent or adherent macrophages at different temperatures (RT vs. 37°C, respectively) and timeframes (from 1 min to few hours following the parasite-cell contact). By using optical tweezers at 37°C, most of the macrophages manipulated oocysts for ~15 min before internalizing them in ~30 min. Variations in internalization kinetics could rely on the way the oocysts were presented to macrophages (their length or width first), as reported for non-biological particles of different geometries (Champion and Mitragotri, 2006). Micropipette aspiration experiments at RT did not provide a robust characterization of the early events of the oocyst phagocytosis by non-adherent macrophages due to the very low number of usable data. Indeed, though oocysts adhered tightly to the macrophage cell membrane following a forced contact, macrophages did not totally internalize oocysts, except few that completed the process as observed at 37°C by using optical tweezers. These results and previous observations (Freppel et al., 2016) indicate that both adherent and non-adherent macrophages are able to internalize oocysts even at a non-physiological temperature. We are currently extending the use of micropipette technique to investigate whether the macrophage senses the oocyst before to capture it as described for a variety of other microbial particles (Heinrich, 2015).

Macrophages treated with latrunculin A (LatA), a depolymerization agent of actin filaments (Fujiwara et al., 2018), were unable to form pseudopods around the oocysts, which remained attached to the macrophage cell membrane. Aside actin, we were further interested in myosin II. It promotes contraction of actin filaments to ensure closure of the phagocytic cup (Barger et al., 2019), and can be enriched in macrophages internalizing rigid particles (Freeman and Grinstein, 2020). As *T. gondii* oocysts are very rigid compared to other biological particles (Dumètre et al., 2013), we investigated whether myosin-II contributed to the internalization of oocysts by treating macrophages with the myosin-II inhibitor blebbistatin (Blebb) (Shu et al., 2005). Blebb-treated macrophages exhibited a fuzzy pattern of their actin cytoskeleton following phalloidin staining, however they internalized oocysts as control ones did. Collectively, our data and previous observations using other inhibitors of the macrophage cytoskeleton (Freppel et al., 2016) suggest a key role of actin polymerization in the formation of the pseudopods and phagocytic cup enclosing the oocysts, and their internalization, as described in classical phagocytosis of beads or biological particles (Rougerie et al., 2013).

Oocyst phagocytosis by RAW macrophages led to the excystation of sporozoites and their differentiation into tachyzoites within 4–6 h following oocyst-macrophage contact, as reported in mice fed with oocysts (Dubey et al., 1997). Interestingly, tachyzoites developed better in macrophages when the oocyst wall was eliminated prior to the internalization assay, either from free sporozoites or free sporocysts. The robust and hermetic oocyst wall is hard to break down by chemical or physical means (Dumètre et al., 2013). In contrast, the sporocyst wall appears more fragile, probably due to the structure of its inner sporocyst wall layer, which is formed of four curved plates held together by thick sutures. If previous studies suggested that the sporocyst wall opens in presence of bile salts at 37°C (Freppel et al., 2019), further investigations are required to characterize its mechanical properties following exposure to various chemical agents.

In conclusion, oocyst phagocytosis by macrophages appears to share common features with classical models of phagocytosis of microbeads. The oocyst wall seems to be the main oocyst substructure that limits the excystation of the sporozoites and therefore their differentiation into tachyzoites. Our oocyst-macrophage *in vitro* platform could further help to address the physical (e.g., contraction of the macrophage cytoskeleton at the phagocytic synapse) and/or chemical factors (e.g., exposure to phagolysosome content) that trigger the opening of the oocyst and the sporocyst walls. *In vivo*, the involvement of phagocytic cells in processing the oocyst walls is uncertain. Such cell types are rare in the intestinal lumen and epithelium, and the presence of mucus could prevent any oocyst-host cell interactions. Only inhalation of oocysts contained in aerosols or dust could involve macrophages at the alveolar level. Such a scenario was suspected following cases of toxoplasmosis by airborne contamination (Teutsch et al., 1979; Lass et al., 2017), but to our knowledge it has never been demonstrated *in vivo*.

## Data Availability Statement

The datasets generated for this study are available on request to the corresponding author.

## Ethics Statement

The animal study was reviewed and approved by Beltsville Area Animal Care and Use Committee (BAACUC), United States Department of Agriculture, Beltsville, MD, USA.

## Author Contributions

P-HP and AD conceived the work. ON, P-HP, CB, LL, SR, JD, and AD carried out the experiments. ON, P-HP, CB, LL, SR, and AD analyzed the data. P-HP, LL, SR, and AD drafted the manuscript. ON, P-HP, LL, SR, NA, JD, and AD reviewed the manuscript.

## Conflict of Interest

The authors declare that the research was conducted in the absence of any commercial or financial relationships that could be construed as a potential conflict of interest.
