# Supplementary material for: Dynamics of Toxoplasma gondii Oocyst Phagocytosis by Macrophages
Source: Front Cell Infect Microbiol. 2020 May 19;10:207. doi: 10.3389/fcimb.2020.00207 (PMC7248298; doi:10.3389/fcimb.2020.00207)
Supplement: Supplementary file 3 [file Data_Sheet_1.pdf]

Supplementary Material

Dynamics of *Toxoplasma gondii* oocyst phagocytosis by macrophages

Omar Ndao,[1,2#] Pierre-Henri Puech,[3,4,5] Camille Bérard,[1,2†] Laurent Limozin,[3,4,5] Sameh Rabhi,[1,2] Nadine Azas,[1,2] Jitender P. Dubey,[6] and Aurélien Dumètre[1,2]*

[1] Aix Marseille Univ, IRD, AP-HM, SSA, VITROME, Marseille, France
[2] IHU-Méditerranée Infection, Marseille, France
[3] Aix Marseille Univ, LAI UM 61, Marseille, F-13288, France
[4] Inserm, UMR_S 1067, Marseille, F-13288, France
[5] CNRS, UMR 7333, Marseille, F-13288, France
[6] United States Department of Agriculture, Agricultural Research Service, Beltsville Agricultural Research Center, Animal Parasitic Diseases Laboratory, Building 1001, Beltsville, MD 20705-2350, USA

*** Correspondence:**
Aurélien Dumètre
aurelien.dumetre@univ-amu.fr

# Present address:
Institut de Biologie Intégrative de la Cellule (i2BC)
Bâtiment 21
Avenue de la Terrasse
91190 Gif-sur-Yvette Cedex

† Deceased 01/14/2017

Supplementary Movie 1: Manipulation of a *Toxoplasma gondii* oocyst by using optical tweezers to initiate a contact with a RAW264.7 macrophage at 37°C. The laser allowed a fine and controlled manipulation of a remote oocyst until contact with the membrane of a macrophage cell (t = 30 sec). Laser was maintained for an additional 10-sec duration prior to be cut off (t = 40 sec). Scale bar = 10 µm. Movie runs at 4 frames per second.

Supplementary Figure 1: Zoom of Figure 1 showing the kinetics of *Toxoplasma gondii* oocyst internalization by RAW264.7 macrophages measured for 6 oocyst-macrophage pairs (P1 to P6) using optical tweezers over the 0-60 min period following initial contact. d0 is the apparent distance measured between the oocyst and macrophage centers at t=0 (i.e. initial contact, laser off) and d the apparent oocyst-macrophage distance at t>0.

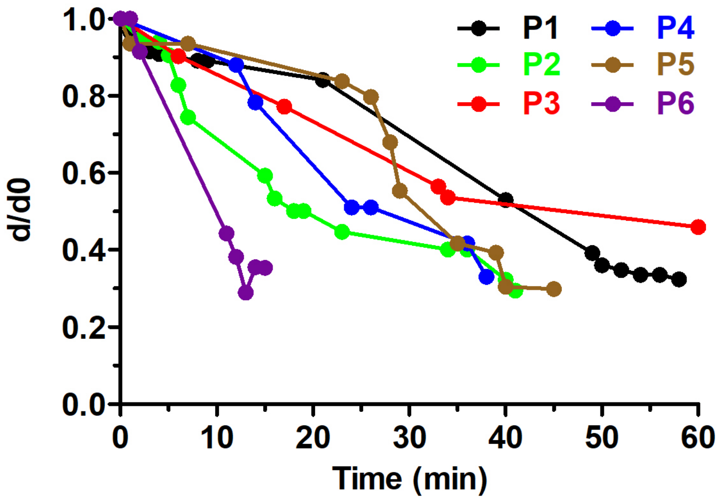

Supplementary Movie 2: Follow-up of the complete internalization of a *Toxoplasma gondii* oocyst by a RAW264.7 macrophage at room temperature by using a micropipette aspiration technique. The oocyst was presented and gently pressed to the macrophage for 1 min. Aspiration was then quickly released on the oocyst, and the holding pipette removed while the macrophage/oocyst pair was observed over time. Images were acquired every minute and analyzed using Fiji software. Oocyst internalization was completed at t=36 min. Scale bar = 10 µm. Movie runs at 4 frames per second.

Supplementary Figure 2: Internalization parameters of a *Toxoplasma gondii* oocyst by a RAW264.7 macrophage at room temperature by using a micropipette aspiration technique. (A) Oocyst-macrophage pair (captured from Supplementary Movie 2) at t=0, 30, and 42 min following oocyst-macrophage initial contact. (B) Quantification of the d/d0 over time, with d0 the apparent distance measured between the oocyst and macrophage centers at t=0 (i.e. oocyst released from its micropipette) and d the apparent oocyst-macrophage distance at t>0. The apparent surface of the oocyst-macrophage contact up to full internalization (t=35 min) zone was calculated as described previously (Freppel et al., 2016). Note that from t=36 to t=42 min, the surface of the contact zone was assimilated to the apparent ellipsoidal surface of the internalized oocyst.

(A)

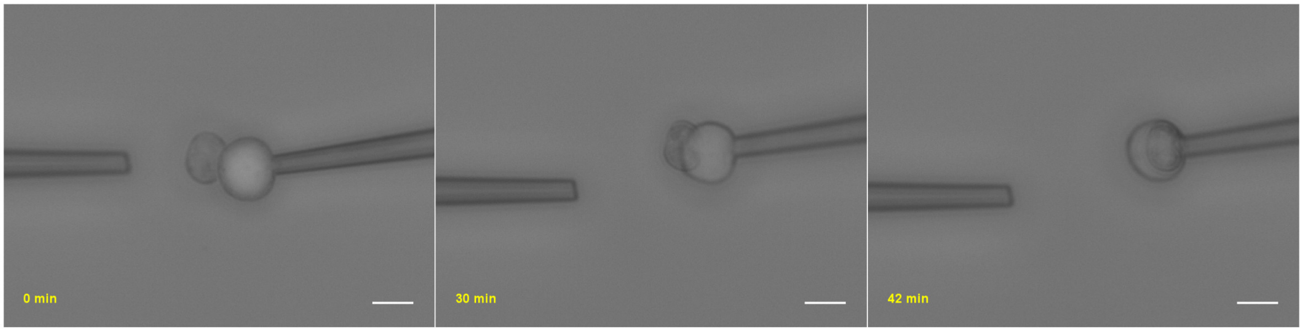

(B)

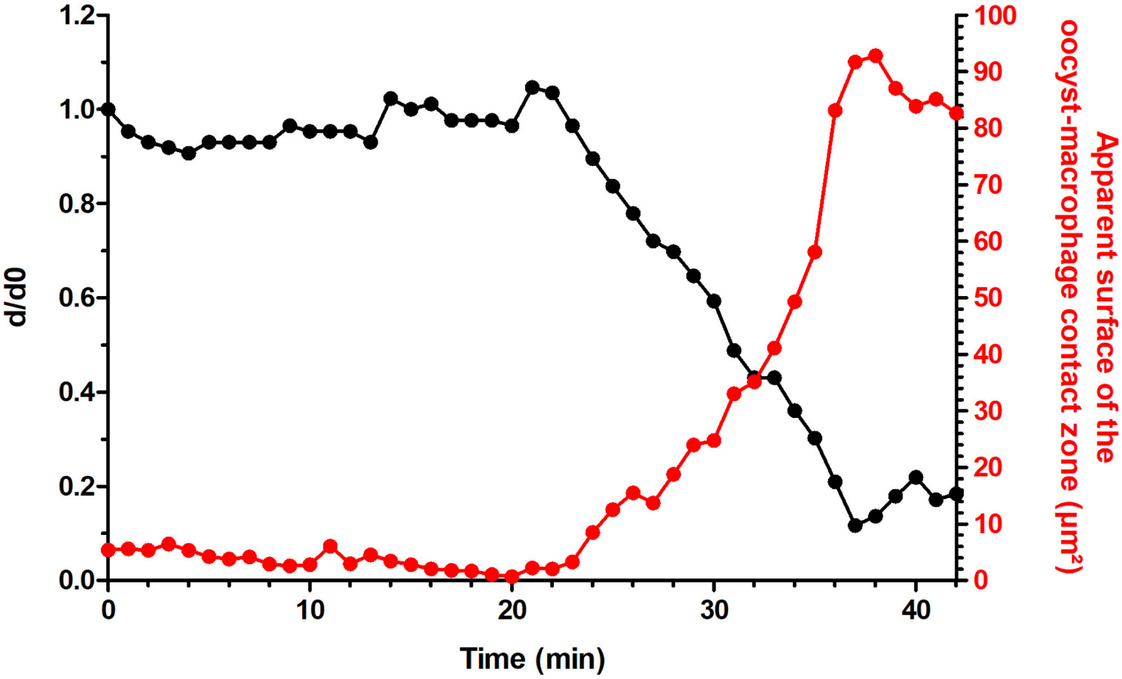

Supplementary Figure 3: Comparison of the oocyst internalization dynamics by RAW264.7 macrophages by using optical tweezers (OT) and micropipette aspiration (MP). Each dot represents an oocyst-macrophage pair. The red line represents the median of data distribution. NS, not significant ($p>0.05$, Student t test).

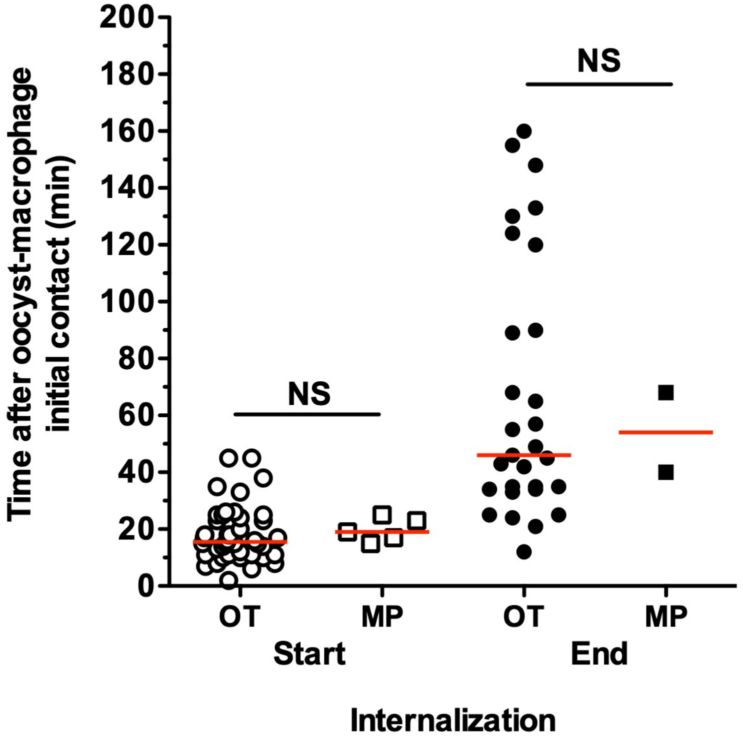

Reference

Freppel, W., Puech, P.-H., Ferguson, D. J. P., Azas, N., Dubey, J. P., and Dumètre, A. (2016). Macrophages facilitate the excystation and differentiation of *Toxoplasma gondii* sporozoites into tachyzoites following oocyst internalisation. *Sci Rep* 6, 33654. doi:10.1038/srep33654.
